# Incidence, aetiology, and sequelae of viral meningitis in UK adults: a multicentre prospective observational cohort study

**DOI:** 10.1016/S1473-3099(18)30245-7

**Published:** 2018-09

**Authors:** Fiona McGill, Michael J Griffiths, Laura J Bonnett, Anna Maria Geretti, Benedict D Michael, Nicholas J Beeching, David McKee, Paula Scarlett, Ian J Hart, Kenneth J Mutton, Agam Jung, Guleed Adan, Alison Gummery, Wan Aliaa Wan Sulaiman, Katherine Ennis, Antony P Martin, Alan Haycox, Alastair Miller, Tom Solomon, UK Meningitis Study Investigators, Adekola Adedeji, Ajdukiewicz Katharine, Birkenhead David, Blanchard Thomas, Cadwgan Antony, Chadwick David, Cheesbrough John, Cooke Richard, Croall John, Crossingham Iain, Dunbar James, Ellis Simon, Faris Camelia, Flegg Peter, Graham Clive, Gray Katherine, Hammersley Shirley, Jones Kevin, Jones Matthew, Kustos Ildiko, Larkin Susan, Mahawish Karim, Maxwell Sarah, Minton Jane, Mohandas Kavya, Mostert Martin, Moran Ed, Murphy Christopher, Pasztor Monika, Paraiso Hassan, Premchand Nikhil, Rathur Haris, Roberts Mark, Robinson Amy, Rosser Andrew, Schumacher Stefan, Silverdale Monty, Stanley Philip, Todd Neil, Watt Alastair, Wiselka Martin

**Affiliations:** a Institute of Infection and Global Health, University of Liverpool, Liverpool, UK; b National Institute for Health Research Health Protection Research Unit on Emerging and Zoonotic Infections, University of Liverpool, Liverpool, UK; c Institute of Translational Medicine, University of Liverpool, Liverpool, UK; d Institute of Psychology, Health and Society, University of Liverpool, Liverpool, UK; e Management School, University of Liverpool, Liverpool, UK; f Royal Liverpool and Broadgreen University Hospitals NHS Trust, Liverpool, UK; g Leeds Teaching Hospitals NHS Trust, Leeds, UK; h Alder Hey Children's NHS Foundation Trust, Liverpool, UK; i The Walton Centre NHS Foundation Trust, Liverpool, UK; j Liverpool School of Tropical Medicine, Liverpool, UK; k Central Manchester Foundation Trust, Manchester, UK; l University of Manchester, Manchester, UK; m North Cumbria University Hospitals NHS Trust, Cumbria, UK

## Abstract

**Background:**

Viral meningitis is increasingly recognised, but little is known about the frequency with which it occurs, or the causes and outcomes in the UK. We aimed to determine the incidence, causes, and sequelae in UK adults to improve the management of patients and assist in health service planning.

**Methods:**

We did a multicentre prospective observational cohort study of adults with suspected meningitis at 42 hospitals across England. Nested within this study, in the National Health Service (NHS) northwest region (now part of NHS England North), was an epidemiological study. Patients were eligible if they were aged 16 years or older, had clinically suspected meningitis, and either underwent a lumbar puncture or, if lumbar puncture was contraindicated, had clinically suspected meningitis and an appropriate pathogen identified either in blood culture or on blood PCR. Individuals with ventricular devices were excluded. We calculated the incidence of viral meningitis using data from patients from the northwest region only and used these data to estimate the population-standardised number of cases in the UK. Patients self-reported quality-of-life and neuropsychological outcomes, using the EuroQol EQ-5D-3L, the 36-Item Short Form Health Survey (SF-36), and the Aldenkamp and Baker neuropsychological assessment schedule, for 1 year after admission.

**Findings:**

1126 patients were enrolled between Sept 30, 2011, and Sept 30, 2014. 638 (57%) patients had meningitis: 231 (36%) cases were viral, 99 (16%) were bacterial, and 267 (42%) had an unknown cause. 41 (6%) cases had other causes. The estimated annual incidence of viral meningitis was 2·73 per 100 000 and that of bacterial meningitis was 1·24 per 100 000. The median length of hospital stay for patients with viral meningitis was 4 days (IQR 3–7), increasing to 9 days (6–12) in those treated with antivirals. Earlier lumbar puncture resulted in more patients having a specific cause identified than did those who had a delayed lumbar puncture. Compared with the age-matched UK population, patients with viral meningitis had a mean loss of 0·2 quality-adjusted life-years (SD 0·04) in that first year.

**Interpretation:**

Viruses are the most commonly identified cause of meningitis in UK adults, and lead to substantial long-term morbidity. Delays in getting a lumbar puncture and unnecessary treatment with antivirals were associated with longer hospital stays. Rapid diagnostics and rationalising treatments might reduce the burden of meningitis on health services.

**Funding:**

Meningitis Research Foundation and UK National Institute for Health Research.

## Introduction

As the incidence of bacterial meningitis decreases, the proportion of meningitis cases caused by viruses is increasing.^1^ The use of molecular diagnostics has also led to increased recognition of neurological viral infections.^2^ The number of reports of viral meningitis and encephalitis in England and Wales was seven times higher in 2013 than in 2004.^2^ Enteroviruses and herpesviruses are commonly reported causes of viral meningitis in adults, but their relative incidences vary in different countries. Finland reports a high incidence of herpesvirus meningitis, whereas Spain has a predominance of enteroviruses.^3^, ^4^

Identification of the cause of meningitis is important to improve clinical care, including reducing unnecessary use of antibiotics and antivirals. Patients with suspected viral meningitis are often treated with antibiotics while a diagnosis of bacterial meningitis is excluded, which results in patients receiving long courses of antibiotics and can extend their hospital stay.^5^ Although aciclovir, which has good in-vitro activity against many herpesviruses, is effective in encephalitis caused by herpes simplex virus and varicella zoster virus, its role in acute meningitis caused by these viruses has never been determined.^6^ Aciclovir has no activity against enteroviruses. Viral meningitis is traditionally considered a benign, self-limiting illness,^7^ but several reports suggest that this might not be the case.^8^, ^9^, ^10^

Recent trends in bacterial, fungal, and mycobacterial meningitis in the UK have been published,^11^ but the clinical burden of viral meningitis remains unknown. We, therefore, did an observational study of adults admitted with suspected meningitis to determine the incidence, causes, and sequelae of viral meningitis.

**Research in context**

**Evidence before this study**

In the past 10–15 years, viral meningitis has been recognised increasingly, and can be a substantial cause of morbidity. Since the widespread introduction of conjugate vaccines against *Haemophilus influenzae* type b in 1992, *Neisseria meningitidis* serogroup C in 1999, and *Streptococcus pneumoniae* in 2002, the incidence of community-acquired bacterial meningitis has been declining. This decline, in combination with increased molecular testing, means that viruses are growing in relative importance as a cause of meningitis. Studies using historical data have also suggested changes in the causes of childhood viral meningitis over several decades.

We searched PubMed for “viral” AND “meningitis” AND “adults”, with no date or language restrictions. 307 publications were returned, of which 22 were cohort studies of the cause of meningitis. Several papers describe the varying causes of meningitis, but only one attempted to determine the incidence, in a cohort of Israeli soldiers. Another study attempted to report the national incidence of viral meningitis in the UK, but it only included laboratory-confirmed cases and did not distinguish between meningitis and encephalitis, the causes, treatment, and prognoses of which are vastly different. No UK study has examined the incidence and causes of viral meningitis in adults. The outcomes following viral meningitis are also unclear, although subtle sequelae such as neurocognitive and sleep disorders have been described.

**Added value of this study**

This study takes a unique approach that combines the benefits of a prospective clinical epidemiological study with laboratory-confirmed cases to estimate the incidence, causes, and sequelae of viral meningitis in UK adults. It is the largest clinical study of adults with viral meningitis reported to date and gives the first accurate incidence of viral meningitis, other causes, and those with no known cause. It also describes the substantial long-term impact that viral meningitis has on quality of life, especially in regard to memory and mental health.

**Implications of all the available evidence**

Our findings demonstrate that viruses are the predominant cause of adult meningitis in the UK, with enteroviruses and herpesviruses responsible for the majority of cases for which a cause is found. Combined with previous studies, this study shows that considerable geographical variation exists in the cause of viral meningitis across the world. We highlight the burden that viral meningitis imposes on the health system and suggest areas in which improvements could be made; a reduction in the length of hospital stays and an increase in meningitis cases with a causal diagnosis might be achieved through more rapid diagnostics. Additionally, we add to the literature suggesting that viral meningitis has effects long after the patient has been discharged.

## Methods

### Study design and participants

In this multicentre, prospective, observational cohort study, patients were recruited from 42 hospitals throughout England, including all 24 acute hospitals in the northwest region of England. Patients were eligible if they were aged 16 years or older, had clinically suspected meningitis, and either underwent a lumbar puncture or, if lumbar puncture was contraindicated, had clinically suspected meningitis and an appropriate pathogen identified either in blood culture or on blood PCR. Individuals with ventricular devices were excluded. Case definitions are in table 1.

**Table 1:** Case definitions

	**Definitions**
Meningitis	Patient with symptoms consistent with meningitis and a CSF leucocyte count >4 × 10^6^ cells per L*†
Viral meningitis	Meningitis and either positive CSF PCR for a viral pathogen or detection of an appropriate pathogen by either throat swab, rectal swab, or serology‡
Bacterial meningitis	Meningitis and detection of an appropriate pathogen from either blood or CSF by PCR, culture, or Gram stain. Or, patient with symptoms consistent with meningitis (who did not have a lumbar puncture) and detection of an appropriate pathogen from blood by PCR, culture, or Gram stain
Lymphocytic meningitis of unknown cause	Meningitis, CSF lymphocytes >50% of total leucocyte count, and no cause identified
Neutrophilic meningitis of unknown cause	Meningitis, CSF neutrophils ≥50% of total leucocyte count, and no cause identified
Undifferentiated meningitis	Meningitis, no CSF leucocyte differential test was performed, and no cause identified
Encephalitis	Altered consciousness for >24 h (including lethargy, irritability, or a change in personality) with no other cause found and two or more of the following signs: fever or history of fever (≥38°C) during the current illness; seizures or focal neurological signs (with evidence of brain parenchyma involvement); CSF pleocytosis (>4 × 10^6^ cells per L); EEG suggesting encephalitis; and neuroimaging suggestive of encephalitis (CT or MRI; adapted from Venkatesan and colleagues^12^)
Tuberculous meningitis	Identification of *Mycobacterium tuberculosis* in the CSF or treated as tuberculous meningitis for ≥2 months
Fungal meningitis	Identification of fungus in the CSF with clinically suspected meningitis
Meningitis, other cause	Meningitis with a cause other than meningeal infection identified

CSF=cerebrospinal fluid. EEG=electroencephalogram.

* Corrected for CSF red blood cell count by 1:700.

† Patients with bacterial and fungal meningitis who had symptoms consistent with meningitis and a pathogen identified in their CSF were classified as having meningitis even if there was no CSF pleocytosis.

‡ Cytomegalovirus, Epstein-Barr virus, and HIV serology.

Written informed consent was obtained from patients. A personal consultee declaration was obtained from friends, carers, or relatives if a patient lacked capacity. Clinical data were recorded on a secure online database (OpenClinica; Waltham, MA, USA). Ethical approval was given by the North Wales Multicentre Research Ethics Committee (reference 11/WA/0218). Research governance approval was given at each hospital. The study protocol can be accessed online.

### Estimation of meningitis incidence

Incidence rates were estimated by dividing the number of patients recruited in the northwest sites, in 1 year, by the total adult population of the same region. To estimate how many cases of meningitis had been missed in the prospective study, we did a retrospective review of laboratory records, spanning the first year of recruitment for each hospital, in four hospitals within the northwest (representing the variation in recruitment rates throughout the whole study—ie, the four hospitals included those with the highest, middle, and lowest recruitment rates). We identified cerebrospinal fluid (CSF) samples with a leucocyte count of more than 4 × 10^6^ cells per L from laboratory records and classified them according to pathogen identified (or unknown if none was found). We applied a proportional inflation, based on the total number of cases (those recruited and those missed) divided by the actual number recruited into the northwest sites in the prospective study, to the initial estimated northwest incidence data. This estimate was then used to calculate the population-standardised number of cases in the UK. Using population data from the Office of National Statistics, the northwest adult population in mid-2012 was 11% of the UK population. Therefore, the national incidence data were derived by assuming that the incidence in the northwest was 11% of the national incidence.

### Outcomes

We assessed outcomes nationally. Clinical outcomes recorded included inpatient mortality and admission to a critical care unit. Patient-reported outcome measures were quality of life, neuropsychological functioning, and symptom resolution. Quality of life was measured using the EuroQol EQ-5D-3L^13^ and 36-Item Short Form Health Survey (SF-36),^14^ both of which are internationally validated. We also recorded the Aldenkamp and Baker neuropsychological assessment schedule (ABNAS)^15^ score and the total morbidity score, which was outlined in Desmond and colleagues' study.^16^ Both of these scores were developed for neurological disorders—namely, epilepsy and meningitis (see appendix for questionnaires). EQ-5D-3L, SF-36, and ABNAS were assessed at 6, 12, 24, and 48 weeks after admission. The total morbidity score recorded resolution of symptoms for 3 weeks after admission. Quality-adjusted life-years (QALYs) were calculated from the EQ-5D-3L. Population-level data for ABNAS are not available, so questionnaires were sent to family or friends of the patient to act as a control group.

### Microbiological testing

All CSF samples were examined by microscopy and culture. CSF PCR was done in the admitting hospitals, regional diagnostic centres, or the University of Liverpool (UK), to test for herpes simplex virus types 1 and 2, varicella zoster virus, and enteroviruses, as well as *Streptococcus pneumoniae* and *Neisseria meningitidis*, following national recommendations.^17^

### Statistical analysis

We used *t* tests to analyse normally distributed continuous data. We applied appropriate transformations in the case of non-normally distributed continuous data. If the transformed data were still not normally distributed, we used Mann-Whitney *U* or Kruskal-Wallis tests. We analysed categorical data by use of χ^2^ or Fisher's exact test. We calculated 95% CIs using Byar's method.^18^ To obtain a 95% CI for the UK incidence, we applied a proportional inflation to the northwest data based on the retrospective data collection. We used logistic regression to assess the association between time to lumbar puncture and getting a microbiologically proven diagnosis. We obtained the SF-6D, a single unit preference-based measure, from the SF-36, and we used non-parametric Bayesian analysis with permission from the University of Sheffield, UK.^19^, ^20^ We applied a Bonferroni correction to the ABNAS domains, and a p value of less than 0·008 was considered statistically significant; last observation carried forward was used for missing data. We determined variables associated with symptom resolution in univariate analyses using log-rank tests. We analysed data using SPSS, version 21.

### Role of the funding source

The funders of the study had no role in study design, data collection, data analysis, data interpretation, or writing of the report. The corresponding author had full access to all the data in the study and had final responsibility for the decision to submit for publication.

## Results

**Figure 1:**
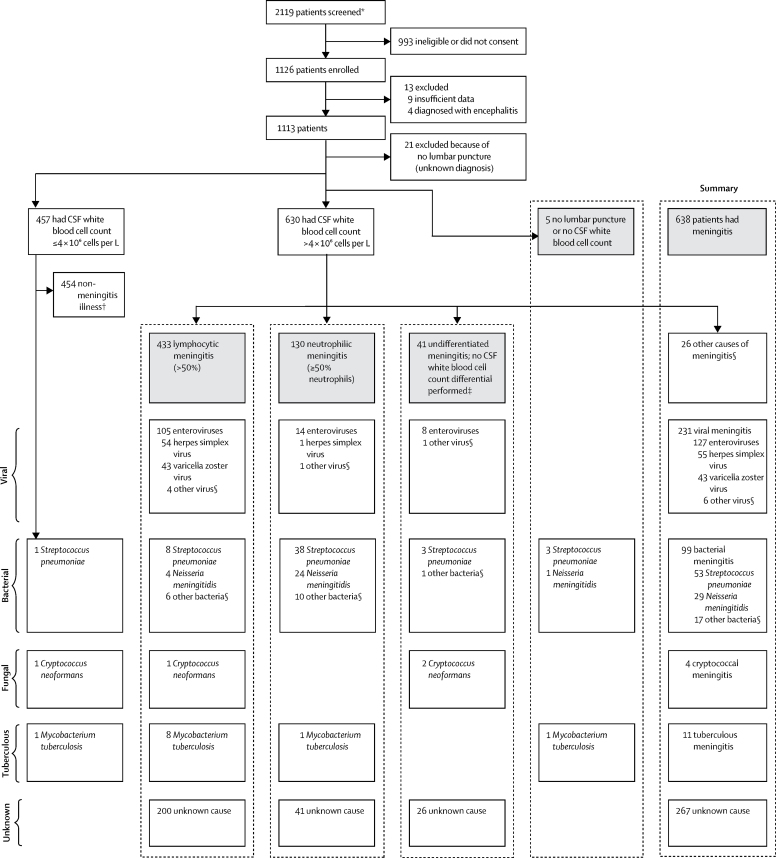
Flow chart of study and final diagnoses of patients recruited CSF=cerebrospinal fluid. *Patients were screened on receipt of a CSF sample in the laboratory. The majority of patients screened and subsequently not recruited did not have meningitis in the differential diagnosis. Mostly, patients had a lumbar puncture to rule out subarachnoid haemorrhage. †Of the non-meningitis cases, 123 were non-specified viral illnesses, 18 were urinary tract infections, 94 were other infections, 95 were headaches or migraines, and 124 were other or unknown illnesses. ‡Median CSF leucocyte count was 12·5 × 10^6^ cells per L (IQR 6–46), and 22 of 41 patients had a leucocyte count of <10 × 10^6^ cells per L. §See table 2 for more details.

1126 patients were enrolled between Sept 30, 2011, and Sept 30, 2014, from throughout England, with 1113 included in the analysis (figure 1). 638 (57%) of 1126 patients fitted the meningitis case definition (table 1). The cause was shown to be viral in 231 (36%) of 638 patients, and bacterial in 99 (16%) of 638 patients (table 2). Enteroviruses were the most frequent viruses, accounting for 55% of all viral meningitis cases (127 of 231 cases), being the single most common cause, accounting for 20% of all meningitis (127 of 638 cases). 101 (44%) of 231 cases were caused by herpesviruses (herpes simplex virus type 2 [n=52], varicella zoster virus [n=43], herpes simplex virus type 1 [n=3], Epstein-Barr virus [n=2], and cytomegalovirus [n=1]). *Streptococcus pneumoniae* was the most common bacterial cause, responsible for 53 (54%) of 99 bacterial cases, but only 8% of all meningitis cases. There were 29 cases of meningococcal meningitis (16 [55%] serogroup B, eight [28%] Y, one [3%] W, and four [14%] unknown serogroup). Four patients had cryptococcal meningitis (all HIV positive), and 11 had tuberculous meningitis. 267 (42%) of 638 patients with meningitis had no cause identified, and, of these patients, 200 (75%) had a lymphocytic CSF (>50% lymphocytes), which was classified as lymphocytic meningitis of unknown cause and 41 (15%) had neutrophil predominance (≥50% neutrophils), which was classified as neutrophilic meningitis of unknown cause. The pre-dominant leucocyte type was unknown in 26 (10%) of 267 patients with no identified cause. Clinical features are shown in table 3.

**Table 2:** Cause of meningitis in UK adults

		**Aetiology of meningitis in UK adults (n=638)**
Viral		
	Enteroviruses	127 (20%)
	Herpes simplex virus type 2	52 (8%)
	Varicella zoster virus	43 (7%)
	Herpes simplex virus type 1	3 (1%)
	Epstein-Barr virus	2 (<1%)
	Cytomegalovirus	1 (<1%)
	Measles	1 (<1%)
	Mumps	2 (<1%)
Bacterial		
	*Streptococcus pneumoniae*	53 (8%)
	*Neisseria meningitidis*	29 (5%)
	*Haemophilus influenzae*	5 (1%)
	*Listeria monocytogenes*	3 (1%)
	*Streptococcus pyogenes*	1 (<1%)
	*Streptococcus agalactiae*	1 (<1%)
	*Streptococcus oralis*	1 (<1%)
	*Mycoplasma pneumoniae*	1 (<1%)
	*Fusobacterium* spp	1 (<1%)
	*Escherichia coli*	1 (<1%)
	*Pseudomonas* spp and *Klebsiella* spp	1 (<1%)
	Positive 16S PCR with no product identified	2 (<1%)
Mycobacterial		
	*Mycobacterium tuberculosis*	11 (2%)
Fungal		
	*Cryptococcus neoformans*	4 (1%)
Infectious causes originating outside the CNS		
	Neurosyphilis	2 (<1%)
	Endocarditis with cerebral emboli or epidural collection	2 (<1%)
	Infected spinal stimulator	1 (<1%)
	Subdural empyema	1 (<1%)
Non-infectious causes of CSF pleocytosis		
	Cerebral haemorrhage	3 (1%)
	Cerebral infarct	2 (<1%)
	Idiopathic intracranial hypertension	2 (<1%)
	Malignancy	2 (<1%)
	Post-surgical	2 (<1%)
	Cluster headache	1 (<1%)
	Epidural haematoma	1 (<1%)
	Lymphocytosis hypophysitis	1 (<1%)
	Migraine	1 (<1%)
	Miller Fisher syndrome	1 (<1%)
	Multiple sclerosis	1 (<1%)
	Neurosarcoidosis	1 (<1%)
	Seronegative uveomeningeal syndrome	1 (<1%)
	Sjogren's syndrome	1 (<1%)
Unknown cause		267 (42%)

Data are n (%). CSF=cerebrospinal fluid.

**Table 3:** Clinical features of study population by cause of meningitis

		**All meningitis**				**Bacterial meningitis**				**Viral meningitis**						**Unknown cause**	
		All patients (n=1117)	Not meningitis (n=454)	All meningitis (n=637)	p value*	All bacterial meningitis (n=99)	Pneumococcal meningitis (n=53)	Meningococcal meningitis (n=28)	p value	All viral meningitis (n=231)	Enteroviral meningitis (n=127)	HSV meningitis (n=55)	VZV meningitis (n=43)	p value†	p value‡	Purulent meningitis (n=41)	Lymphocytic meningitis (n=199)
Age (years)		34 (25·0–49·0)	36 (25·0–48·0)	34 (25·0–49·0)	0·788	56 (34·0–65·0)	60 (42·5–65·5)	44 (19·5–57·0)	0·002	32 (24·0–42·0)	30 (24·0–36·0)	34 (26·0–50·0)	37 (25·0–53·0)	0·004	<0·001	33 (23·0–48·5)	33 (27·0–45·5)
Sex					0·065				0·15					0·01	0·006		
	Male	413 (37%)	152 (33%)	249 (39%)	..	50 (51%)	24 (45%)	17 (61%)	..	79 (34%)	48 (38%)	10 (18%)	19 (44%)	..	..	17 (41%)	71 (36%)
	Female	704 (63%)	302 (67%)	388 (61%)	..	49 (49%)	29 (55%)	11 (39%)	..	152 (66%)	79 (62%)	45 (82%)	24 (56%)	..	..	24 (59%)	128 (64%)
Neck stiffness		603/1079 (56%)	238/436 (55%)	348/616 (56%)	0·571	39/92 (42%)	19/47 (40%)	11/29 (38%)	0·83	149/229 (65%)	80/126 (64%)	43/54 (80%)	22/42 (52%)	0·01	<0·001	20/36 (56%)	100/179 (56%)
Headache		1025/1096 (94%)	415/446 (93%)	587/623 (94%)	0·445	82/92 (89%)	43/47 (91%)	26/29 (90%)	1	229/231 (99%)	127/127 (100%)	54/54 (100%)	42/43 (98%)	0·19	<0·001	36/41 (88%)	190/197 (96%)
Photophobia		747/1083 (69%)	320/443 (72%)	415/613 (68%)	0·119	39/91 (43%)	18/47 (38%)	14/29 (48%)	0·39	185/231 (80%)	111/127 (87%)	42/55 (76%)	28/43 (65%)	0·004	<0·001	20/35 (57%)	121/178 (68%)
History of rash		139/974 (14%)	75/437 (17%)	78/607 (13%)	0·062	21/93 (23%)	5/48 (10%)	14/29 (48%)	<0·001	29/228 (13%)	11/125 (9%)	6/54 (11%)	11/43 (26%)	0·02	0·03	2/33 (6%)	14/175 (8%)
Confusion		217/1077 (20%)	65/436 (15%)	145/615 (24%)	<0·001	54/95 (57%)	36/50 (72%)	10/29 (34%)	0·001	22/227 (10%)	10/125 (8%)	5/53 (9%)	7/43 (16%)	0·28	<0·001	12/38 (32%)	35/194 (18%)
Sore throat		189/1048 (18%)	109/427 (26%)	77/594 (13%)	<0·001	12/90 (13%)	4/46 (9%)	5/28 (18%)	0·285	31/221 (14%)	22/124 (18%)	6/50 (12%)	1/41 (2%)	0·04	0·936	8/36 (22%)	23/189 (12%)
Vomiting		601/1088 (55%)	229/441 (52%)	359/622 (58%)	0·061	62/94 (66%)	28/48 (58%)	24/29 (83%)	0·03	123/229 (54%)	66/126 (52%)	26/54 (48%)	29/43 (67%)	0·14	0·051	24/39 (62%)	118/196 (60%)
Diarrhoea		107/1049 (10%)	42/429 (10%)	63/596 (11%)	0·684	17/92 (18%)	6/47 (13%)	5/29 (17%)	0·59	25/220 (11%)	13/120 (11%)	4/53 (8%)	7/42 (17%)	0·4	0·093	4/33 (12%)	14/190 (7%)
Myalgia		363/1029 (35%)	173/420 (41%)	182/585 (31%)	0·001	21/90 (23%)	4/46 (9%)	13/29 (45%)	<0·001	73/221 (33%)	38/124 (31%)	22/51 (43%)	9/40 (23%)	0·1	0·127	16/36 (44%)	57/179 (32%)
Genital ulcers		8/941 (1%)	3/369 (1%)	5/550 (1%)	0·878	0/88 (0)	0/44 (0)	0/29 (0)	NA	5/206 (2%)	0/112 (0)	5/48 (10%)	0/40 (0)	0·001	0·188	0/32 (0)	0/167 (0)
Seizures		46/1069 (4%)	25/432 (6%)	20/613 (3%)	0·048	8/96 (8%)	6/51 (12%)	1/29 (3%)	0·41	0/226 (0)	0/126 (0)	0/51 (0)	0/43 (0)	NA	<0·001	4/39 (10%)	3/189 (2%)
History of meningitis		117/1077 (11%)	44/437 (10%)	72/615 (12%)	0·396	11/95 (12%)	9/50 (18%)	1/29 (3%)	0·08	24/226 (11%)	7/126 (6%)	15/53 (28%)	2/41 (5%)	<0·001	0·894	2/39 (5%)	24/193 (12%)
Fever (>38°C)		260/1117 (23%)	110/454 (24%)	143/618 (23%)	0·511	39/99 (39%)	26/53 (49%)	7/29 (24%)	0·03	43/226 (19%)	28/127 (22%)	8/55 (15%)	6/43 (14%)	0·33	<0·001	8/38 (21%)	39/193 (20%)
Positive Kernig's sign		104/472 (22%)	51/203 (25%)	49/259 (19%)	0·113	9/25 (36%)	4/12 (33%)	2/7 (29%)	1	27/116 (23%)	14/70 (20%)	11/31 (35%)	2/11 (18%)	0·269	0·242	1/17 (6%)	7/78 (9%)
Positive Brudzinski's sign		30/184 (16%)	11/72 (15%)	18/108 (17%)	0·839	4/12 (33%)	2/6 (33%)	1/3 (33%)	1	10/41 (24%)	5/26 (19%)	5/10 (50%)	0/4 (0)	0·123	0·712	0/11 (0)	3/34 (9%)
Glasgow Coma Scale		15 (15–15)	15 (15–15)	15 (15–15)	0·807	14 (10–15)	11 (9–14)	15 (14–15)	<0·001	15 (15–15)	15 (15–15)	15 (15–15)	15 (15–15)	0·25	<0·001	15 (15–15)	15 (15–15)
White blood cell count (× 10^9^ cells per L)		9·4 (7·1–12·9)	9·3 (6·8–12·9)	9·45 (7·4–13·0)	0·252	16·4 (12·5–21·9)	16·9 (13·7–21·5)	17·8 (11·1–24·4)	0·74	8·8 (7·1–10·6)	8·8 (6·9–10·6)	9·4 (7·9–12·0)	8·6 (6·4–10·3)	0·07	<0·001	9·6 (7·9–13·9)	8·9 (7·1–11·8)
C-reactive protein (mg/L)		50 (22–122)	55 (28–121)	43 (19–123)	0·034	164 (67–261)	169 (69–263)	184 (111–295)	0·34	20 (15–38)	20 (16–39)	11 (10–28)	26 (19–76)	0·02	<0·001	38 (15–148)	31 (18–82)
C-reactive protein <10 mg/L		453/1047 (41%)	163/428 (38%)	278/596 (47%)	0·006	6/93 (6%)	5/49 (10%)	0/27 (0)	0·15	122/210 (55%)	42/119 (35%)	44/53 (83%)	37/41 (90%)	<0·001	<0·001	10/38 (26%)	105/183 (57%)
CSF opening pressure (cm CSF)		20 (15–26)	18 (15–21)	22 (16–28)	1	30 (21–40)	36 (26–40)	30 (18–35)	0·07	21 (16·–27)	21 (15–26)	22 (20–29)	25 (16–30)	0·34	<0·001	24 (21–30)	20 (15–25)
CSF leucocyte count (× 10^6^ cells per L)		77 (5–306)	NA	155 (44–450)	<0·001	1800 (377–4850)	2180 (668–4340)	2000 (480–7175)	0·81	188 (67–355)	118 (44–218)	374 (225–718)	249 (106–450)	<0·001	<0·001	133 (29–730)	102 (34–255)
CSF neutrophil percentage		5 (0–37)	NA	10 (0–47)	<0·001	90 (66–95)	90 (68–96)	90 (79–98)	0·62	5 (0–14)	8 (2–22)	1 (0–10)	0 (0–10)	<0·001	<0·001	80 (60–90)	4 (0–10)
CSF protein (g/L)		0·53 (0·32–0·98)	0·32 (0·25–0·45)	0·81 (0·53– 1·38)	<0·001	4·00 (2·00–6·68)	5·63 (3·10–8·12)	3·00 (1·17–6·67)	0·03	0·76 (0·54–1·12)	0·57 (0·45–0·75)	1·14 (0·90–1·32)	1·18 (0·89–1·40)	<0·001	<0·001	0·80 (0·50–1·44)	0·68 (0·49–1·00)
CSF glucose (mmol/L)		3·2 (2·8–3·7)	3·5 (3·2–3·9)	3·0 (2·5–3·5)	<0·001	1·1 (0·3–2·7)	0·5 (0·2–1·7)	1·1 (0·4–2·8)	0·02	3·0 (2·7–3·4)	3·1 (2·8–3·5)	3·0 (2·7–3·4)	2·9 (2·5–3·2)	0·009	<0·001	3·3 (2·7–3·9)	3·1 (2·8–3·4)
CSF:serum glucose ratio		0·58 (0·46–0·67)	0·63 (0·57–0·70)	0·52 (0·40–0·62)	<0·001	0·12 (0·03–0·41)	0·04 (0·01–0·26)	0·15 (0·05–0·42)	0·02	0·56 (0·49–0·63)	0·58 (0·53–0·64)	0·52 (0·48–0·61)	0·54 (0·45–0·63)	0·104	<0·001	0·57 (0·41–0·66)	0·57 (0·46–0·66)

Data are median (IQR) for continuous data and n/N (%) evaluable for categorical data. CSF=cerebrospinal fluid. HSV=herpes simplex virus. VZV=varicella zoster virus. NA=not applicable.

* Significance values comparing not meningitis and all meningitis.

† Significance values comparing enteroviral, HSV, and VZV meningitis cases.

‡ Significance values comparing all bacterial meningitis and all viral meningitis cases.

732 patients were recruited from the northwest sites throughout the whole duration of the study. Using both the prospective and retrospective data from the northwest sites, the incidence of viral meningitis was estimated to be 2·73 per 100 000 per year and that of bacterial meningitis 1·24 per 100 000 per year in UK adults (table 4). When all cases were considered, including those with no identified cause, the annual incidence of all meningitis in UK adults was 13·47 per 100 000.

**Table 4:** Estimated incidence of community acquired meningitis in UK adults by cause of meningitis

	**Patients recruited in northwest sites over duration of study**	Estimated number of patients in the northwest in 1 year*	**Estimated annual incidence per 100 000 population (95% CI) in northwest**†**based on numbers recruited**	**Proportional inflation**‡	**Estimated annual corrected incidence per 100 000 population (95% CI)**	**Estimated number of cases a year in the UK (95% CI)**
Enteroviral meningitis	85	39	0·70 (0·49–0·95)	2·3	1·57 (1·11–2·14)	802 (567–1091)
Herpes simplex virus meningitis	38	18	0·31 (0·19–0·51)	2·5	0·78 (0·48–1·27)	399 (242–647)
Varicella zoster virus meningitis	29	13	0·24 (0·12–0·40)	1·5	0·36 (0·19–0·59)	182 (94–303)
Total confirmed viral meningitis	154	71	1·27 (0·99–1·60)	2·2	2·73 (2·13–3·44)	1389 (1084–1750)
*Streptococcus pneumoniae* meningitis	26	13	0·23 (0·12–0·39)	4·5	1·04 (0·53–1·73)	529 (268–884)
*Neisseria meningitidis* meningitis	15	7	0·12 (0·04–0·25)	1·0	0·12 (0·04–0·25)	63 (23–125)
Total confirmed bacterial meningitis	47	22	0·39 (0·24–0·58)	3·2	1·24 (0·76–1·87)	631 (390–951)
Meningitis of unknown cause	176	81	1·45 (1·15–1·80)	7·3	10·58 (8·4–13·14)	5390 (4277–6695)
All meningitis§	385	178	3·17 (2·72–3·67)	4·3	13·47 (11·55–15·59)	6864 (5886–7944)

* Based on sites recruiting patients for a median duration of 26 months (IQR 19–32).

† Calculated using Office of National Statistics mid-2012 population data and the northwest having 11% of the UK population.

‡ Based on number of cases missed in 1 year in northwest sentinel sites.

§ Includes unknown cause and causes other than bacteria and viruses.

**Figure 2:**
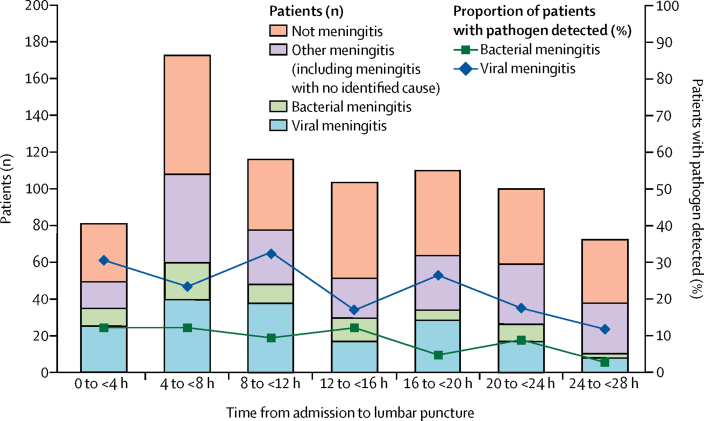
Number of patients with a proven microbiological diagnosis following delay in lumbar puncture Lines represent the percentage of all patients with a pathogen detected. Bars represent actual numbers of patients. Data beyond 28 h are not shown because of small numbers.

901 (81%) of 1113 patients had neurological imaging, with the majority (776 [70%] of 1113) before lumbar puncture. Only 90 (12%) of these 776 patients had an indication for imaging before lumbar puncture, as recommended in national guidelines (ie, at least one of Glasgow Coma Scale ≤12, uncontrolled seizures, papilloedema, or focal neurological signs).^21^ The most common indications were a score of 12 or less on the Glasgow Coma Scale in 54 (7%) of 776 patients and seizures in 36 (5%) of 776 patients; five (1%) patients had papilloedema and eight (1%) had focal neurological findings. The median time from admission to antibiotics was 2 h (IQR 0–10 [n=23]) in patients who did not have imaging before lumbar puncture compared with 3 h (1–11 [n=563]) in those who did (p=0·004), and the median time from admission to lumbar puncture was 8 h (3–22 [n=299]) in patients who did not have imaging versus 18 h (9–30 [n=776]) in those who did (p<0·001). The median time from admission to lumbar puncture was longer in patients with lymphocytic meningitis of unknown cause (21 h [IQR 9–38]) than in those with proven viral meningitis (13 h [7–23]; p<0·001), proven bacterial meningitis (13 h [5–23]; p<0·001), and neutrophilic meningitis of unknown cause (15 h [7–23]; p=0·008). The median time to lumbar puncture for all patients was 17 h (IQR 8–29). The chances of having a pathogen detected in viral meningitis was reduced by 1% for every hour of delay in lumbar puncture after admission (odds ratio [OR] 0·988 [95% CI 0·982–0·995]; p=0·001; figure 2). For bacterial meningitis, there was also a reduction of 1% for each hour delay, but this was not statistically significant (0·995 [0·989–1·002]; p=0·16). 24 (24%) of 99 patients with bacterial meningitis were diagnosed by molecular methods alone. The role of the different tests in diagnosing bacterial meningitis is shown in the appendix.

139 (60%) of 231 patients with viral meningitis had at least one dose of an antiviral (aciclovir or valaciclovir), and 51 (37%) of 139 received a course, defined as at least 5 days. 42 (43%) of 98 patients with herpes simplex virus or varicella zoster virus meningitis received a course of antivirals with a median duration of 10 days (IQR 5–30). The treatment regimen varied considerably between patients with herpes simplex virus and varicella zoster virus meningitis (appendix). Patients in whom no cause was identified were more likely to receive antiviral drugs than those in whom a definitive diagnosis of enterovirus meningitis was made (50 [20%] of 248 *vs* eight [6%] of 127; p=0·001). The antiviral drugs would have been ineffective against enteroviruses. Most patients (160 [69%] of 231) with proven viral meningitis also received at least one dose of antibiotics (median duration 1 day [IQR 0–3]), and 199 (75%) of 267 patients without an identified cause received at least a single dose. 328 (72%) of 454 patients who did not have meningitis received empirical antibiotics.

The median length of stay for patients with viral meningitis was 4 days (IQR 3–7). Patients with herpesvirus meningitis stayed in hospital longer than did patients with enteroviral meningitis (median 6 days [IQR 4–10] *vs* 3·5 days [3–5]; p<0·001), and those with varicella zoster virus meningitis stayed longer than did those with herpes simplex virus (8 days [IQR 5–11] *vs* 5 days [3–8]; p=0·02). Patients who received antivirals were in hospital longer than those who did not (median 9 days [IQR 6–12] *vs* 3 days [2–5]; p<0·001), and individuals with lymphocytic meningitis of unknown cause stayed in hospital slightly longer than did those with proven viral meningitis (5 days [3·0–8·5] *vs* 4 days [3–7]; p=0·09). Seven (1%) of 1113 patients died before discharge, five (71%) of whom had meningitis (three had pneumococcal, one had tuberculous, and one had malignant meningitis). 91 (8%) of 1113 patients required admission to intensive care; 52 (57%) of 91 patients had bacterial meningitis, and 37 (71%) of those 52 had pneumococcal disease. No patients with viral meningitis died or required admission to critical care.

**Figure 3:**
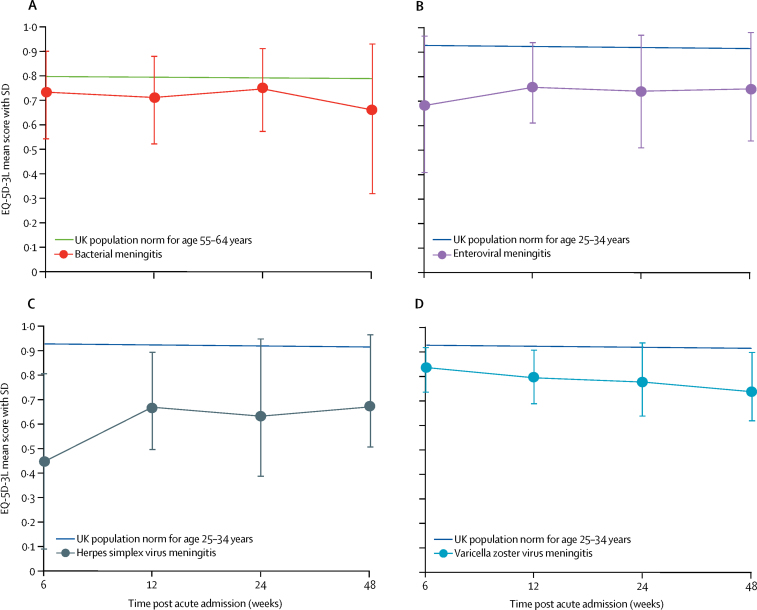
EQ-5D-3L scores over time from acute illness for meningitis The EQ-5D-3L determines health status across five domains, which is converted into a utility score. A score of 1 represents best health and 0 represents dead. Health states perceived to be worse than death have a negative score. Datapoints are mean scores, and error bars indicate SDs. Median age for viral meningitis was 32 years (IQR 24–42), and median age for bacterial meningitis was 56 years (34–65).

Quality of life was reduced in all groups at all timepoints, when compared with the UK population, especially in those aged 25–35 years (figure 3). EQ-5D-3L utility scores were similar for patients with both viral and bacterial meningitis; all patients had scores worse than the population norms for the relevant age groups. They were significantly lower for patients with herpes simplex virus meningitis, compared with the other viral causes, at 6 weeks after discharge (mean score 0·45 [SD 0·36] *vs* 0·72 [0·25]; p=0·004). 12 (86%) of 14 patients with herpes simplex virus meningitis who returned the questionnaires had problems with anxiety or depression at 6 weeks (appendix). Supporting, and confirming, the EQ-5D-3L data, all groups had worse SF-6D scores than UK norms (appendix). The mean QALYs for patients with viral meningitis, over the first year, was 0·72 (SD 0·04). Compared with the age-matched UK population, patients with viral meningitis had a mean loss of 0·2 QALYs (SD 0·04) in that first year (appendix). There was no significant difference in median time to resolution of headache between patients with viral and those with bacterial meningitis, as measured by the total morbidity score (7 days *vs* 8 days, p=0·09; appendix). Patients with viral meningitis had significantly worse ABNAS scores than did 234 healthy controls at all four timepoints in the year after illness (appendix).

## Discussion

This study provides the first estimate of the incidence of viral meningitis in UK adults. Using clinical and laboratory data, we estimated the annual incidence of confirmed viral meningitis in UK adults to be 2·73 per 100 000. Previous UK studies of meningitis have been based on coding data or laboratory reports, which exclude cases that have no identified cause.^1^, ^2^, ^11^ We estimated the annual incidence of all meningitis to be 13·47 per 100 000. Previously, a similar estimate of the annual incidence of meningitis in the USA was 27·9 per 100 000,^22^ although the study is 20 years old and included adults and children. The incidence is likely to be substantially lower now, given the impact of immunisation.^23^

Enteroviruses were the most common cause, accounting for just above 50% of all confirmed viral meningitis cases. Herpesviruses accounted for 101 (44%) of 231 cases, substantially more than in previous studies from other countries.^4^ This finding might, in part, be explained by different rates of herpes simplex virus type 2 seroprevalence, which is higher in northern than in southern Europe.^24^

In line with other studies, a substantial proportion of our patients had no cause identified,^3^, ^4^ which poses a challenge as to how to categorise them. There have been several attempts at diagnostic algorithms, each of which has its limitations and none of which has become routine clinical practice.^25^ We chose a pragmatic and objective classification, which is used in UK hospitals daily, based on predominant CSF leucocyte type. We recognise that this classification does not equate to presumed viral or bacterial meningitis, and indeed 18% of patients with bacterial meningitis had a lymphocytic CSF and 7% of viral meningitis cases (mostly enteroviral) had a neutrophil predominance. Nevertheless, this method is a helpful way of providing an initial patient classification. Patients with lymphocytic meningitis of unknown cause had a significantly longer time from admission to lumbar puncture than those with proven viral or bacterial meningitis and those with neutrophilic meningitis of unknown cause, suggesting that an early lumbar puncture might increase the number of patients having a cause identified. It could be, as is the case for enterovirus meningitis, that there is a change in the immune response from neutrophils initially to lymphocytes later.

Diagnosis of a specific virus reduces inappropriate antibiotic usage, length of hospital stay, and hospital admission costs.^5^, ^7^ We have also shown that it reduces the unnecessary use of antivirals. 21% of patients with lymphocytic meningitis of unknown cause received a course of aciclovir or valaciclovir compared with 6% of patients diagnosed with enteroviral meningitis, on which aciclovir would have no effect. With no evidence base to support aciclovir treatment in meningitis caused by herpes simplex virus or varicella zoster virus, as has been highlighted previously, there was much variation in practice.^6^ Almost half of the patients in the current study received antivirals, resulting in longer hospital admissions. Most patients who had antivirals had intravenous treatment, necessitating inpatient care. A trial of aciclovir or valaciclovir in acute herpesvirus meningitis would help to determine best practice. Improvement of diagnostic testing so that more patients can have a specific cause determined quickly could reduce unnecessary use of antimicrobials and therefore reduce hospital stays and other investigations.^7^ Full diagnostic accuracy and cost-effectiveness studies should be done before any new tests are adopted widely.

Once viral meningitis is diagnosed, efforts should focus on symptomatic treatment and expediting discharge. Theoretically, diagnosis can happen quickly; a lumbar puncture and the diagnostic PCR can be done within a few hours. However, in our study, the median time from admission to lumbar puncture for all patients was 17 h, and the median length of hospitalisation for patients with viral meningitis was 4 days. The prolonged time from admission to lumbar puncture is concerning. International guidelines all stress the urgency of the diagnostic lumbar puncture;^25^, ^26^, ^27^ delays decrease pathogen yield and can increase mortality.^28^, ^29^, ^30^ The length of time to get a lumbar puncture might explain why a large proportion of patients had no cause identified in our study, especially those with viral meningitis, for which there was a highly significant association between time to lumbar puncture and likelihood of getting a definitive diagnosis. Unnecessary neuroimaging might have contributed to the delays, and has been highlighted previously as a risk factor for increased mortality in bacterial meningitis.^30^, ^31^ In the UK, the requirement for all patients to be transferred out of the emergency department within 4 h creates unintended pressure, causing key investigations such as lumbar puncture to be deferred until patients have been admitted to a ward. Additional delays in diagnosis occur if the CSF sample is sent to an offsite laboratory for analysis. Because of sample batching and transport, it can take several days from doing a lumbar puncture to receiving results, despite the rapidity of the test itself. If PCR is done locally, 7 days a week, on receipt of a single CSF sample, the length of the hospital stay can be reduced to less than a day, resulting in substantial cost savings.^7^ To make this saving relatively simple, changes are required, such as doing lumbar punctures in the emergency department and having diagnostics available on site.

Despite viral meningitis often being referred to as benign and self-limiting,^7^ we found long-term neuropsychiatric sequelae, particularly anxiety, depression, and neurocognitive dysfunction. Although patients with bacterial meningitis have more severe disease initially in terms of critical care need and mortality, over the longer term all patients with meningitis, viral and bacterial, had sequelae affecting quality of life, including significant problems with memory and mental health.

There are limitations to our study. Because of its prospective nature, we risked not recruiting all eligible patients. We accounted for this by identifying missed cases retrospectively in the laboratories and then applying an extrapolation. We extrapolated the incidence from the northwest to the whole country, which assumes that there is minimal variation in incidence throughout the UK. We found the incidence of pneumococcal, meningococcal, and all viral meningitis was similar to that found in other UK-based studies that used only laboratory data.^2^, ^11^ Relying on CSF analysis excluded patients who did not have a lumbar puncture but allowed us to accurately define our cohort. Our definitions might have missed some cases of viral meningitis with a CSF cell count of less than 5 × 10^6^ cells per L or those in whom a lumbar puncture was not done. Children, especially neonates, can have clinical features of meningitis, with viruses detected in the CSF, without a CSF pleocytosis.^32^ This phenomenon is less well recognised in adults. 58% of our patients who had a lumbar puncture had meningitis, which is higher than the proportion found in other studies overseas,^33^ and might indicate a higher threshold for lumbar puncture in the UK than in other countries. Given that we looked only for the most common viruses, we cannot exclude the possibility that other rare, novel, or emerging viruses might have been responsible for some cases. However, previous attempts using novel techniques have not identified significantly more pathogens than routine approaches.^34^

In summary, this study shows that viruses are the major cause of meningitis in adults in the UK, and impose a considerable clinical burden, both acutely and longer term. To improve management and reduce costs, there is a pressing need for better diagnostic practices, including rapid tests and the delivery of high-quality viral diagnostics locally. Treatments also need to be developed and assessed that could allow quicker recovery and fewer longer-term sequelae.
