# Supplementary material for: Incidence, aetiology, and sequelae of viral meningitis in UK adults: a multicentre prospective observational cohort study
Source: Lancet Infect Dis. 2018 Sep;18(9):992–1003. doi: 10.1016/S1473-3099(18)30245-7 (PMC6105576; doi:10.1016/S1473-3099(18)30245-7)
Supplement: Supplementary appendix [file mmc1.pdf]

THE LANCET
Infectious Diseases

Supplementary appendix

This appendix formed part of the original submission and has been peer reviewed. We post it as supplied by the authors.

Supplement to: McGill F, Griffiths MJ, Bonnett LJ, et al. Incidence, aetiology, and sequelae of viral meningitis in UK adults: a multicentre prospective observational cohort study. *Lancet Infect Dis* 2018; published online June 29. http://dx.doi.org/10.1016/S1473-3099(18)30245-7.

Appendix

Table S1. Median number of days for Total Morbidity Score symptoms to resolve* according to differing aetiologies [percentage not achieving resolution of symptoms by 21 days following admission]

	Not meningitis	Viral meningitis	Bacterial meningitis	Lymphocytic meningitis – unknown aetiology	Purulent meningitis – unknown aetiology
**Headache**	7 [27]	7 [14]	8 [14]	11 [18]	9 [33]
**Nausea/vomiting**	3 [4]	3 [0]	4 [5]	4 [0]	5 [0]
**Photophobia**	5 [12]	4 [4]	8 [3]	8 [8]	7 [18]
**Myalgia**	6 [35]	6 [16]	12 [36]	10 [26]	9 [33]
**Neck Stiffness**	7 [22]	5 [17]	8 [20]	10 [13]	8 [0]
**Total Score**	8 [23]	6 [8]	7 [31]	10 [17]	9 [20]

***Duration is until resolution of symptoms to mild according to the Total Morbidity Score (score of 6 or less for total score, 2 or less for headache and 1 or less for all other symptoms) for 2 or more days with no subsequent increase**

Table S2. Aldenkamp and Baker Neuropsychological Assessment Schedule (ABNAS) scores in all domains and totals at 6, 12, 24 and 48 weeks after admission.							
**6 weeks**							
	Fatigue	Motor	Slowing	Concentration	Memory	Language	Total
**Healthy controls (n=234)**	2 (0,5)	1 (0,5)	2 (0,4)	1 (0,3)	0 (0,2)	1 (0,2)	7 (2, 19.75)
**Viral Meningitis (n=67)**	5** (2,8)	0 (0,2)	5** (2,8)	3** (1,6)	4** (2,6)	2** (1,4)	22* (8.5,34.5)
**Bacterial Meningitis (n=33)**	4** (2,7)	1 (0,2)	4** (2,6)	2(0,4)	3**(1.5,4.5)	1(0,2)	17* (9,26)
**Enteroviral meningitis (n=43)**	5** (2,10)	0 (0,2)	5** (1,9)	3** (1,6)	4** (2,7)	2** (0,4)	22.5* (4,37)
**HSV meningitis (n=13)**	7** (4.25,7.75)	1 (0,4.5)	5** (4,7.75)	3** (1,7)	3** (1.5,6)	2 (1,5)	17* (14,36)
**VZV meningitis (n=10)**	4 (1.25,7)	0 (0,1.5)	3 (0,8)	1 (0,4.25)	3** (1, 6.5)	1 (0,2.25)	10 (5,29)
**12 weeks**							
**Healthy controls (n=234)**	2 (0,5)	1 (0,5)	2 (0,4)	1 (0,3)	0 (0,2)	1 (0,2)	7 (2, 19.75)
**Viral Meningitis (n=73)**	4** (2,8)	1(0,2)	5** (1,7)	3** (1,6)	4** (1,6)	2** (1,4)	18* (6.75,31.5)
**Bacterial Meningitis (n=49)**	3 (1,7)	1 (0,2)	3 (1.25,5)	2 (0,4)	3** (1,6)	2 (0,4)	13* (7,26.25)
**Enteroviral meningitis (n=43)**	4** (2,8)	1 (0,2)	5** (1,8)	4** (1,7)	4** (1,6)	2** (0,4)	23* (6,33)
**Herpes simplex meningitis (n=17)**	6 (2,10)	2 (0,3.5)	5** (2.5, 10)	3.5** (1.25,6.75)	4** (2,6.75)	3** (1,5)	16.5* (10.25,41.5)
**Varicella zoster meningitis (n=12)**	2.5 (1,7.5)	0 (0,1.5)	1.5 (0,6)	1.5 (0,3.75)	3** (1,4)	2 (1.25,3)	12 (4.5,26)
**24 weeks**							
**Healthy controls (n=234)**	2 (0,5)	1 (0,5)	2 (0,4)	1 (0,3)	0 (0,2)	1 (0,2)	7 (2, 19.75)
**Viral Meningitis (n=60)**	4** (1,9)	1(0,2)	5 (1,8)	4** (1,5.75)	3.5** (1,6)	2** (0,4)	17.5* (6.75,28)
**Bacterial Meningitis (n=41)**	3 (2,7)	0** (0,1.5)	3.6 (1,6.75)	2 (0,4)	3** (1.5,5.5)	1 (0,3.5)	14* (6,27)
**Enteroviral meningitis (n=38)**	4.5** (1.75, 8.25)	1 (0,2)	4.5 (1,8)	4** (1,5.25)	3.5** (0.75,6)	1.5 (0,4)	20* (5.5,28)
**Herpes simplex meningitis (n=12)**	5 (2,10)	1.5 (0,3.5)	5.5 (2.5,8.75)	4(0,6.5)	3.5** (1,8.25)	2.5 (0,4.5)	19* (11,33)
**Varicella zoster meningitis (n=9)**	2 (0,9.5)	2 (0,4)	1 (0,5.75)	3 (1,8)	3** (1.5,8)	2 (1,4.5)	10.5 (4.25,33.5)
**48 weeks**							
**Healthy controls (n=234)**	2 (0,5)	1 (0,5)	2 (0,4)	1 (0,3)	0 (0,2)	1 (0,2)	7 (2, 19.75)
**Viral Meningitis (n=46)**	4 (0.5,9.5)	1 (0,3)	3 (1,8.5)	2.5 (0,5.25)	3** (1,5.5)	2 (0,3)	14.5* (4.25, 37.25)
**Bacterial Meningitis (n=17)**	4 (0.5,7)	0 (0,3)	4 (0.5,5.5)	1(0,4.5)	3** (1,5.5)	1 (0,3.5)	11 (5,27.75)
**Enteroviral meningitis (n=27)**	4 (0,9)	1 (0,3)	4 (0,8)	1 (0,5)	3** (0,5)	1 (0,3)	15 (3, 38)
**Herpes simplex meningitis (n=13)**	3 (1,10)	1.5 (0,4)	2.5 (1.25,8.5)	2 (0,7.5)	2.5** (1,7.5)	1.5(0.25,3.75)	8 (5,45)
**Varicella zoster meningitis (n=4)**	7 (3,9.5)	3.5 (1.5,4.75)	8 (2.5,9.75)	4.5 (2.5,7.25)	4.5** (3.25,9.5)	3 (1.5,3.75)	29.5* (16,36.25)

Values are median (IQR). *significant, compared with the healthy controls, at the level of <0.05. **Significance, compared with the healthy controls, at the level of <0.008 (corrected for multiple testing using a Bonferroni correction). Control subjects only completed the questionnaire once. No significant differences were seen between the total scores for each of the viral aetiologies.

If the cases with missing data were excluded, rather than using last observation carried forward, the only data point that becomes non-significant is that of the total score for varicella zoster meningitis at 48 weeks.

Supplementary figures

Figure S1. Diagnostic investigations used in bacterial meningitis

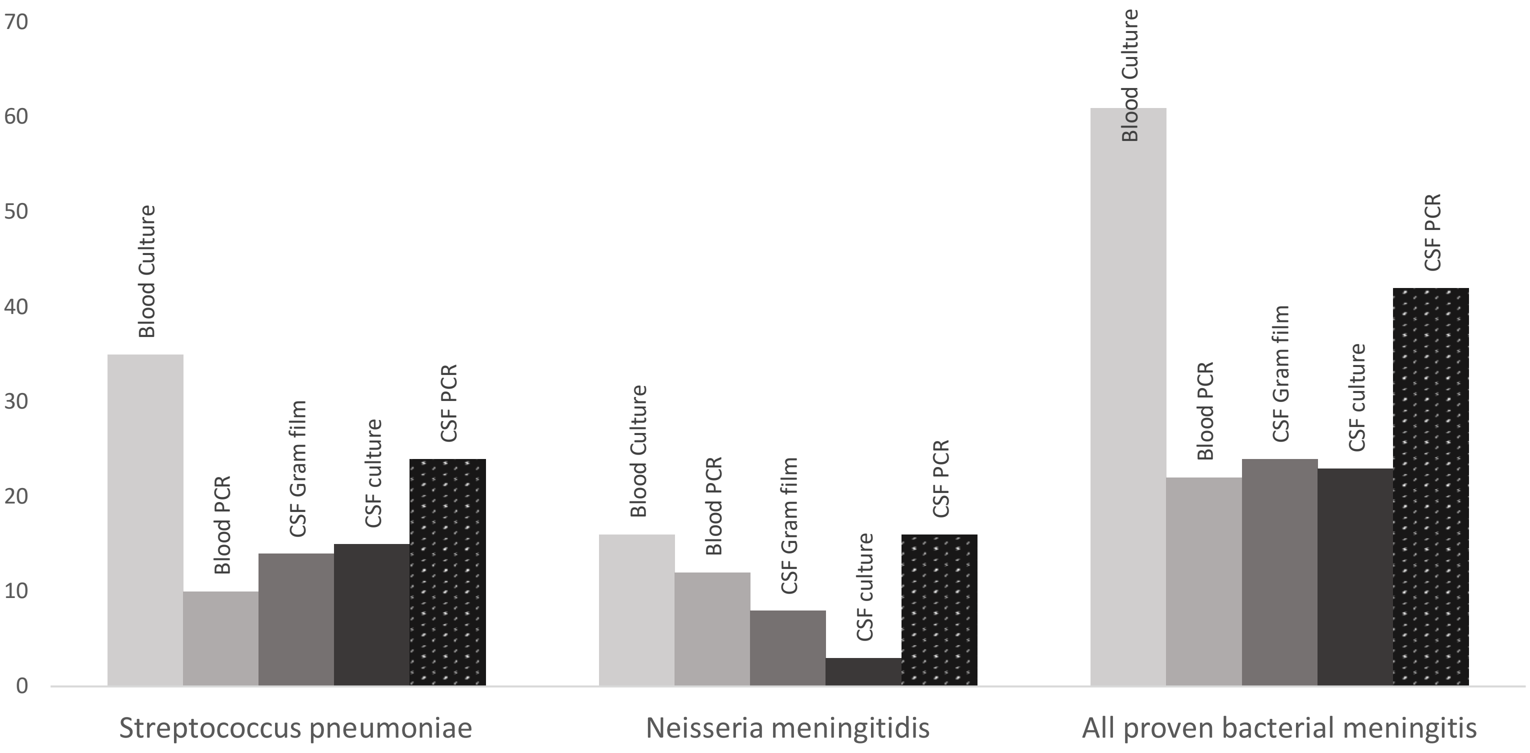

Number of positive investigations for different aetiologies of bacterial meningitis. Some patients had more than one positive diagnostic test. PCR = Polymerase chain reaction. CSF = cerebrospinal fluid.

Figure S2. Variation in antiviral prescribing

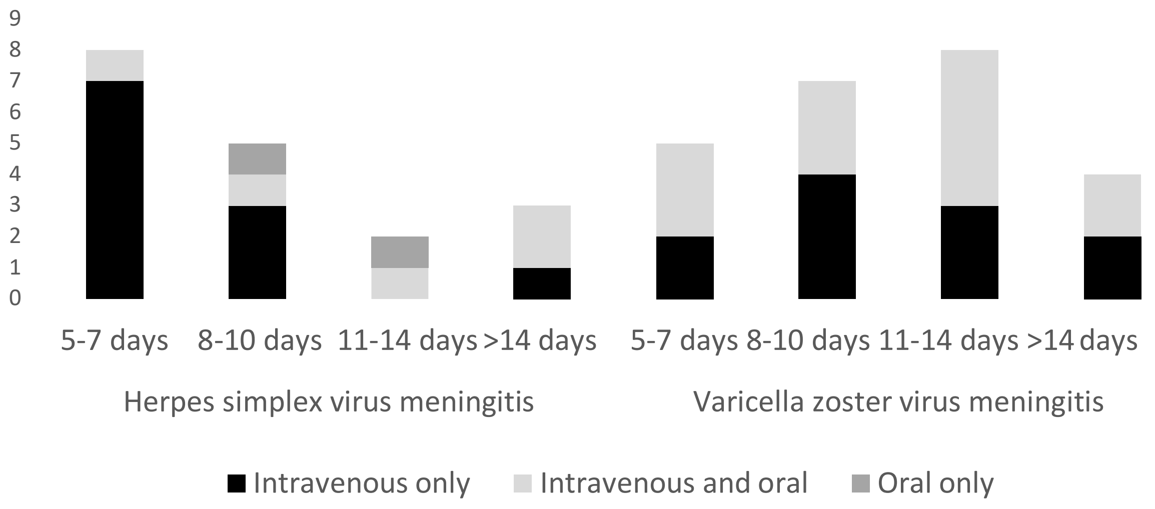

There was no consensus in antiviral prescribing for patients with herpes virus meningitis who were given a course (≥ 5 days) of antivirals. When analysing all patients in the study older age, longer duration of symptoms, the presence of confusion or a lymphocytic CSF were all significantly associated with having a course of antivirals (p = <0.001, 0.007, 0.002 and <0.001 respectively). No other clinical features were associated with giving antivirals. When only patients with herpes meningitis were considered (HSV and VZV) the only significant associations with giving a course of antivirals were the duration of symptoms prior to coming into hospital [median of 79 days versus 48 days for those who were given a course of antivirals compared with those who weren't (p=0.04)] and aetiology. Patients with Varicella zoster meningitis were more likely to have antivirals prescribed than those with herpes simplex meningitis [24 (56%) versus 18 (33%), p=0·026]. No patients with varicella zoster meningitis had only oral treatment.

Figure S3. Percentage of respondents with problems in each of the domains of the EQ5D over time. A) – 6 weeks, b) -12 weeks, c) 24 weeks and d) 48 weeks .

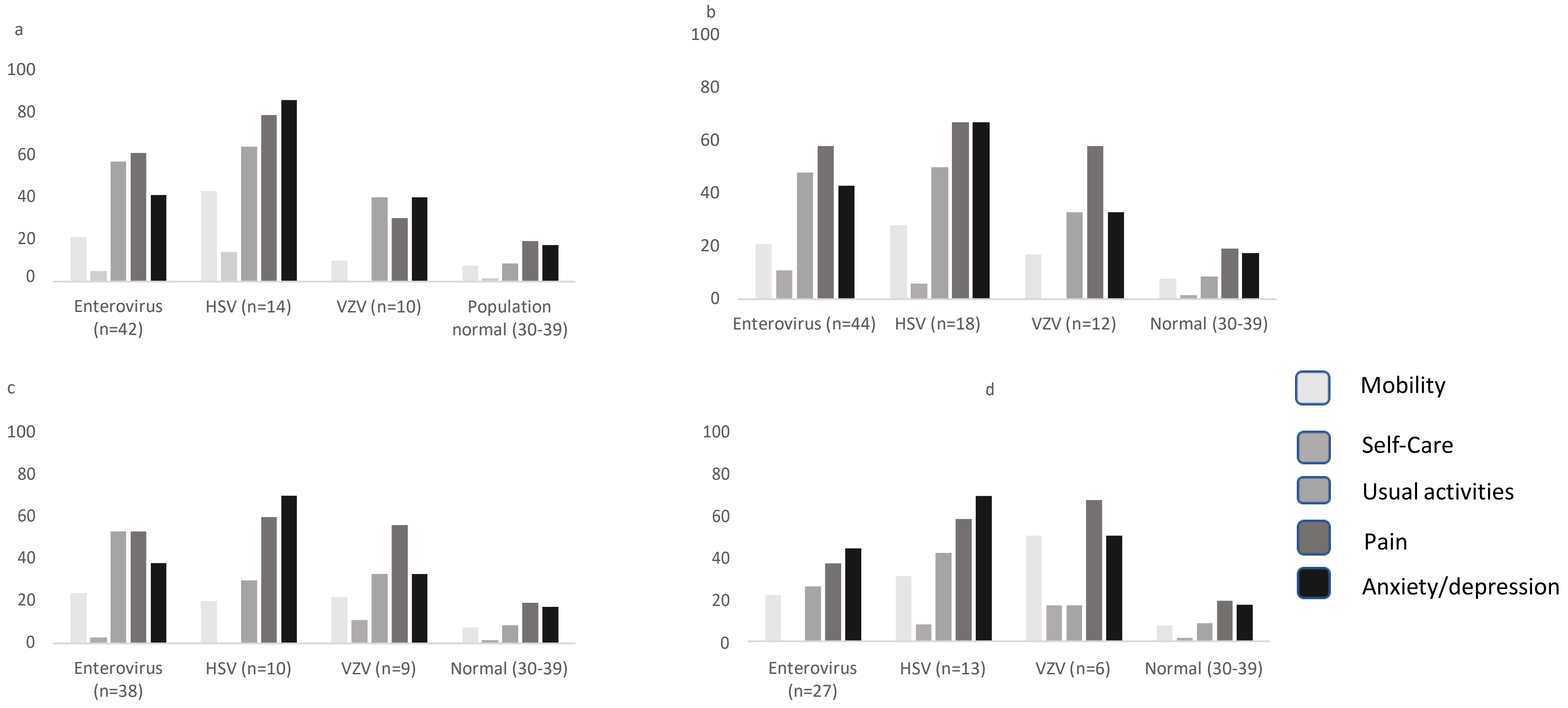

HSV = Herpes simplex virus. VZV = Varicella zoster virus

Figure S4. SF-6D utility scores over time in patients with viral or bacterial meningitis compared with the UK normal population.

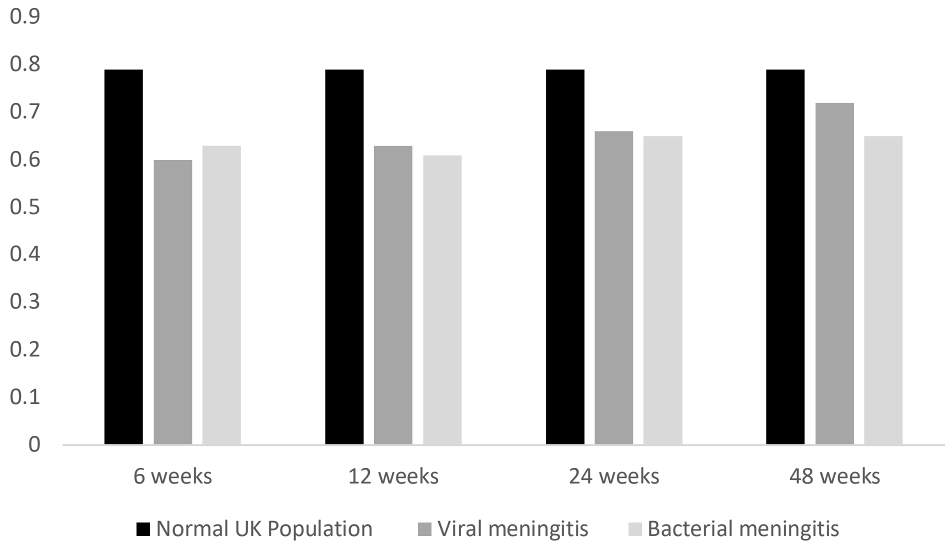

Figure S5. Percentage of patients with viral meningitis with reported difficulties in different domains of the SF6D at a) 6 weeks, b) 12 weeks, c) 24 weeks and d) 48 weeks.

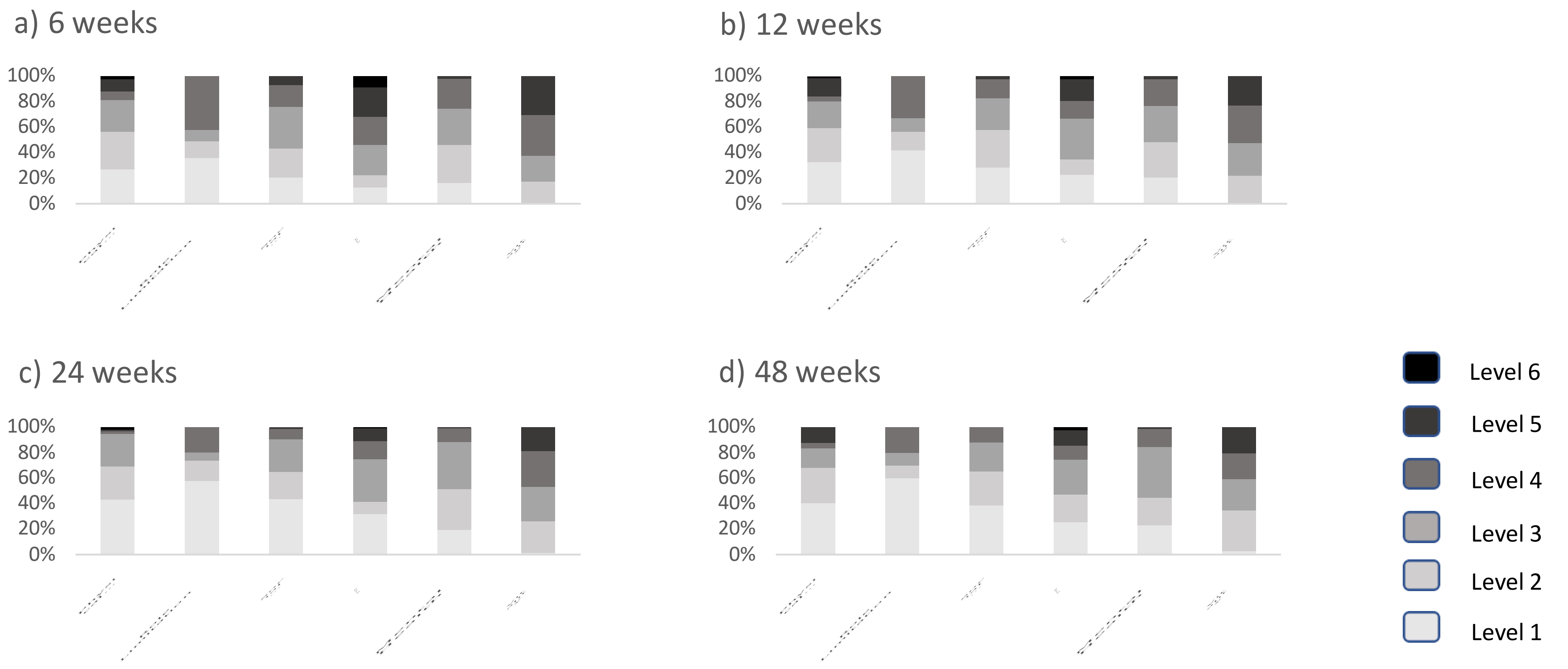

Within the different domains of the SF-36 most patients with viral meningitis had minimal or no problems with physical and social functioning but there was a skew towards poorer health states in the domains of pain, mental health and vitality, supporting the results found in the EQ-5D-3L. Level 6 (darkest grey) represents the worst health state and 1 (lightest), the best. The different domains have a different number of health states; role limitation only has 4 levels, social functioning, mental health and vitality have 5 with the remaining domains having 6.

Figure S6. QALYs lost in the first year after illness in patients with viral meningitis compared with the UK population normal.

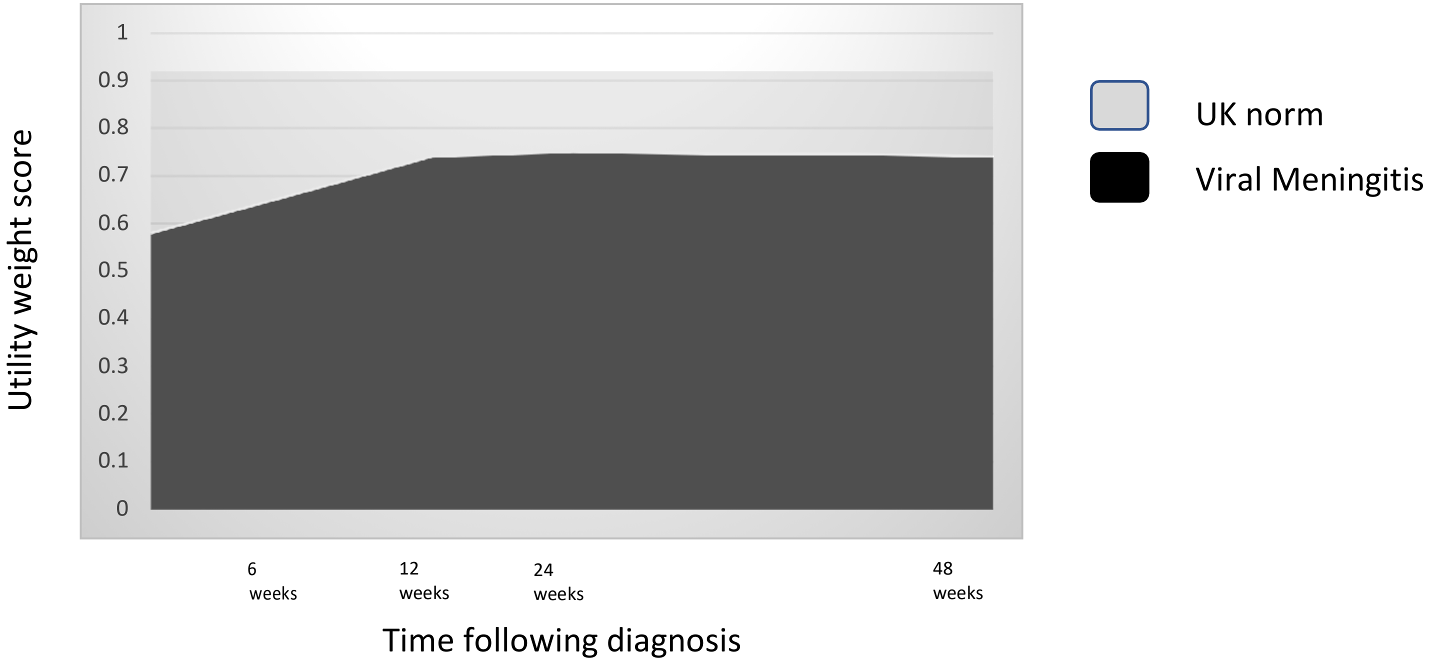

Data were not available for discharge, therefore utility weight scores were estimated using linear extrapolation from the 6 and 12 week data. On analysis of change over time, no further significant changes were detected after 12 weeks, although the utility score was significantly lower than the UK norm at all time points. QALYs lost is shown by the difference in area under the curve.

Figure S7. Total Aldenkamp and Baker Neurological Assessment Scores (ABNAS)

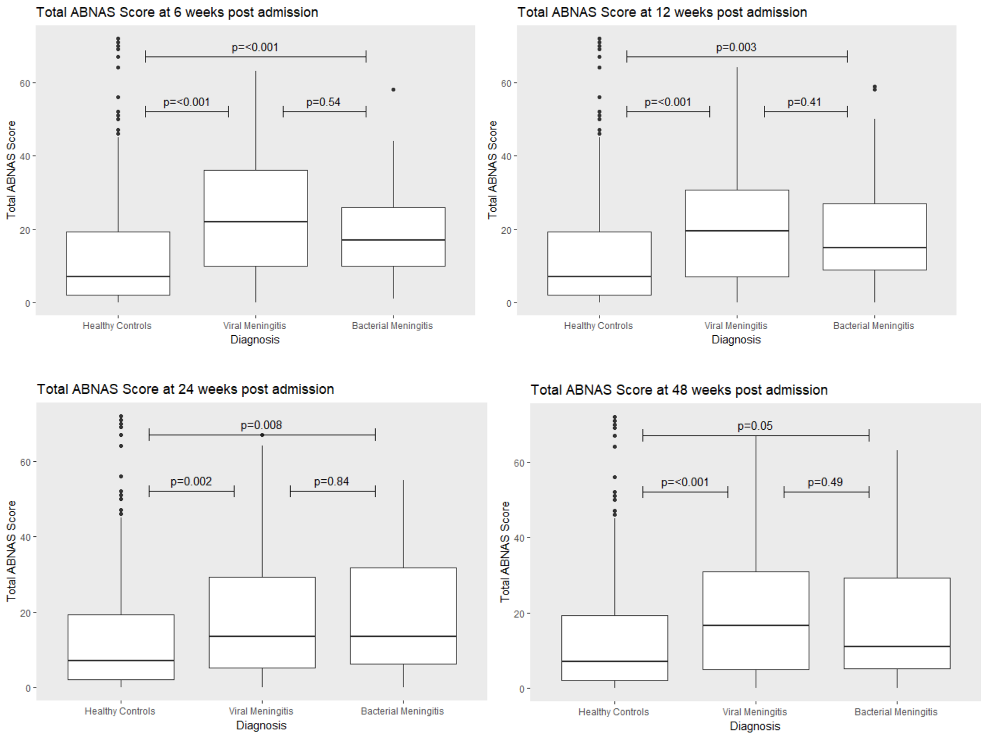

The ABNAS assesses neuropsychological functioning across 6 domains - fatigue, motor, slowing, concentration, memory and language. Higher scores correspond to greater levels of dysfunction, with a worst possible score of 72. There are no normative values for the ABNAS, therefore, healthy controls were recruited from relatives or friends of the patient. The healthy controls had a median total ABNAS score of seven. There were no significant differences between the viral aetiologies.

Total Morbidity Score

This score will help us see how quickly people recover from meningitis and allow us to compare with others who had other illnesses. Please score your symptoms according to the scales below. If you have a total score of 0 for 7 consecutive days then you don't need to fill in any more. If you are discharged from hospital before 21 days please return in the attached addressed envelope (no need for a stamp).

Day 1 = date of admission to hospital.

Headache
0 none
1 barely noticeable
2 mild (able to function)
3 moderate (requires medication)
4 moderately severe (able to function but prefers staying in bed)
5 severe (must stay in bed)
6 very severe (incapacitating)

Nausea and vomiting
0 none
1 mild (nausea present, no vomiting)
2 moderate (2 or fewer episodes of vomiting in the last 24 hours)
3 severe (more than 2 episodes of vomiting in the last 24 hours

Neck Stiffness
0 none
1 mild (able to function)
2 moderate (can bend neck, but significant difficulty)
3 severe (unable to bend neck)

Photophobia (eyes sensitive to the light)
0 none
1 mild (slight sensitivity to light)
2 moderate (can open eyes but would prefer not to)
3 severe (unable to open eyes because too painful)

Myalgia (muscle aches)
0 none
1 mild(noticeable but no need for painkillers
2 moderate(need to take pain killers)
3 severe(uncontrolled with simple painkillers e.g. paracetamol and non steroidals)

Fever
0 <37.5
1 mild (37.5-38)
2 moderate (38-39)
3 severe (>39)

Total Morbidity Score

Subject ID

Day	1	2	3	4	5	6	7	8	9	10
Date										
Time										
Headache										
Nausea/Vomiting										
Neck Stiffness										
Sensitivity of eyes to light										
Muscle aches										
Fever										
Temperature										
Total Score										

Headache 0 none1 barely noticeable 2 mild (able to function)3 moderate (requires medication) 4 moderately severe (able to function but prefers staying in bed) 5 severe (must stay in bed) 6 very severe (incapacitating)

Nausea and vomiting 0 none 1 mild (nausea present, no vomiting)2 moderate (2 or fewer episodes of vomiting in the last 24 hours) 3 severe (more than 2 episodes of vomiting in the last 24 hours

Neck Stiffness 0 none 1 mild (able to function) 2 moderate (can bend neck, but significant difficulty) 3 severe (unable to bend neck)

Photophobia (eyes sensitive to the light)0 none 1 mild (slight sensitivity to light) 2moderate (can open eyes but would prefer not to) 3 severe (unable to open eyes because too painful)

Myalgia (muscle aches)0none 1 mild(noticeable but no need for painkillers 2 moderate(need to take pain killers) 3 severe(uncontrolled with simple painkillers e.g. paracetamol and non steroidals)

Fever 0 <37.5 1 mild (37.5-38) 2 moderate (38-39) 3 severe (>39)

Temperature Please record actual temperature for that day.

Total Morbidity Score

Subject ID

Day	11	12	13	14	15	16	17	18	19	20	21
Date											
Time											
Headache											
Nausea/vomiting											
Neck Stiffness											
Sensitivity of eyes to light											
Muscle aches											
Fever											
Temperature											
Total Score											

Headache 0 none1 barely noticeable 2 mild (able to function)3 moderate (requires medication) 4 moderately severe (able to function but prefers staying in bed) 5 severe (must stay in bed) 6 very severe (incapacitating)

Nausea and vomiting 0 none 1 mild (nausea present, no vomiting)2 moderate (2 or fewer episodes of vomiting in the last 24 hours) 3 severe (more than 2 episodes of vomiting in the last 24 hours

Neck Stiffness 0 none 1 mild (able to function) 2 moderate (can bend neck, but significant difficulty) 3 severe (unable to bend neck)

Photophobia (eyes sensitive to the light)0 none 1 mild (slight sensitivity to light) 2moderate (can open eyes but would prefer not to) 3 severe (unable to open eyes because too painful)

Myalgia (muscle aches)0none 1 mild(noticeable but no need for painkillers 2 moderate(need to take pain killers) 3 severe(uncontrolled with simple painkillers e.g. paracetamol and non steroidals)

Fever 0 <37.5 1 mild (37.5-38) 2 moderate (38-39) 3 severe (>39)

Temperature Please record actual temperature for that day.

Health Questionnaire

(English version)

© 1998 EuroQol Group. EQ-5D™ is a trade mark of the EuroQol Group

By placing a checkmark in one box in each group below, please indicate which statements best describe your own health state today.

Mobility

I have no problems in walking about ❑

I have some problems in walking about ❑

I am confined to bed ❑

Self-Care

I have no problems with self-care ❑

I have some problems washing or dressing myself ❑

I am unable to wash or dress myself ❑

Usual Activities (*e.g. work, study, housework, family or leisure activities)*

I have no problems with performing my usual activities ❑

I have some problems with performing my usual activities ❑

I am unable to perform my usual activities ❑

Pain/Discomfort

I have no pain or discomfort ❑

I have moderate pain or discomfort ❑

I have extreme pain or discomfort ❑

Anxiety/Depression

I am not anxious or depressed ❑

I am moderately anxious or depressed ❑

I am extremely anxious or depressed ❑

To help people say how good or bad a health state is, we have drawn a scale (rather like a thermometer) on which the best state you can imagine is marked 100 and the worst state you can imagine is marked 0.

We would like you to indicate on this scale how good or bad your own health is today, in your opinion. Please do this by drawing a line from the box below to whichever point on the scale indicates how good or bad your health state is today.

Your own health state today

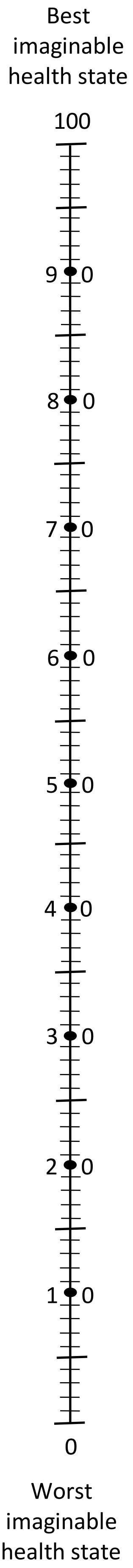

SF-36 QUESTIONNAIRE

(1992 -- Medical Outcomes Trust)

Patient Name: ----------------------------- **Date: ---------------------**

1. In general, would you say your health is: (circle one)

Excellent Very good Good Fair Poor

2. <u>Compared to one year ago</u>, how would you rate your health in general <u>now</u>? (circle one)

Much better now than one year ago. Somewhat better now

than one year ago. About the same as one year ago.

Somewhat worse than one year ago. Much worse than

one year ago.

3. The following items are about activities you might do during a typical day. Does your health now limit you in these activities? If so, how much? (Mark each answer with an **X**)

**ACTIVITIES**	**Yes, Limited A Lot**	**Yes, Limited A Little**	**No, Not Limited At All**
a. **Vigorous activities,** such as running, lifting heavy objects, participating in strenuous sports			
b. **Moderate activities,** such as moving a table, pushing a vacuum cleaner, bowling, or playing golf			
c. Lifting or carrying groceries			
d. Climbing **several** flights of stairs			
e. Climbing **one** flight of stairs			
f. Bending, kneeling or stooping			
g. Walking **more than a mile**			
h. Walking **several blocks**			
i. Walking **one block**			
j. Bathing or dressing yourself			

4. During the past 4 weeks, have you had any of the following problems with your work or other regular daily activities as a result of your physical health? (Mark each answer with an **X**)

	**YES**	**NO**
a. Cut down on the **amount of time** you spent on work or other activites		
b. **Accomplished less** than you would like		
c. Were limited in the **kind** of work or other activities		
d. Had **difficulty** performing the work or other activities (for example, it took extra effort)		

5. During the past 4 weeks, have you had any of the following problems with your work or other regular daily activities as a result of any emotional problems (such as feeling depressed or anxious)? (Mark each answer with an **X**)

	**YES**	**NO**
a. Cut down the **amount of time** you spent on work or other activities		
b. **Accomplished less** than you would like		
c. Didn't do work or other activities as **carefully** as usual		

6. During the past 4 weeks, to what extent has your physical health or emotional problems interfered with your normal social activities with family, friends, neighbors or groups? (circle one)

Not at all Slightly Moderately Quite a bit Extremely

7. How much bodily pain have you had during the past 4 weeks? (circle one)

None Very mild Mild Moderate Severe Very severe

8. During the past 4 weeks, how much did pain interfere with your normal work (including both work outside the home and housework)?

Not at all A little bit Moderately Quite a bit Extremely

9. These questions are about how you feel and how things have been with you during the past 4 weeks. For each question, please give the one answer that comes closest to the way you have been feeling. How much of the time during the past 4 weeks - (Mark each answer with an **X**)

	All of the Time	Most of the Time	A Good Bit of the Time	Some of the Time	A Little of the Time	None of the Time
a. Did you feel full of pep?						
b. Have you been a very nervous person?						
c. Have you felt so down in the dumps that nothing could cheer you up?						
d. Have you felt calm and peaceful?						
e. Did you have a lot of energy?						
f. Have you felt downhearted and blue?						
g. Did you feel worn out?						
h. Have you been a happy person?						
i. Did you feel tired?						

10. During the past 4 weeks, how much of the time has your physical health or emotional problems interfered with your social activities (like visiting with friends, relatives, etc.)? (circle one)

All of the time Most of the time Some of the time A little of the time None of the time

11. How TRUE or FALSE is each of the following statements for you?

	Definitely True	Mostly True	Don't Know	Mostly False	Definitely False
a. I seem to get sick a little easier than other people					
b. I am as healthy as anybody I know					
c. I expect my health to get worse					
d. My health is excellent					

A-B Neuropsychological Assessment Scale

Please fill in the following questionnaire for how you feel today. Please circle the appropriate answers.

For each item, if it is not a problem circle **0**; if it is a mild problem circle **1**; if it is a moderate problem circle **2**; and if it is a serious problem circle **3**. If a question is not relevant for you, no answer should be given

**1**	I am less enthusiastic about day to day activities	0 1 2 3
**2**	My mind does not work as fast as it should	0 1 2 3
**3**	I have difficulties remembering names of people	0 1 2 3
**4**	I have difficulties following a book or a film	0 1 2 3
**5**	I feel clumsy	0 1 2 3
**6**	I have problems finding the correct word	0 1 2 3
**7**	I am less capable of undertaking initiatives	0 1 2 3
**8**	My thinking has slowed down	0 1 2 3
**9**	I forget things, for example an appointment or where I put an object	0 1 2 3
**10**	I have difficulties concentrating on the things I am doing	0 1 2 3
**11**	I cannot use a pen or pencil accurately	0 1 2 3
**12**	I have problems understanding what I read	0 1 2 3
**13**	I tire easily and have little energy	0 1 2 3
**14**	It takes me longer to do day to day things	0 1 2 3
**15**	I forget things that people have said to me	0 1 2 3
**16**	I can't concentrate for more than a short period of time	0 1 2 3
**17**	I constantly bump against tables, doorposts, etc.	0 1 2 3
**18**	I feel worn out	0 1 2 3
**19**	It costs more time for me to get started	0 1 2 3
**20**	I get confused and forget what I was doing	0 1 2 3
**21**	I get distracted more easily	0 1 2 3
**22**	I sometimes stutter or am unable to find the correct words	0 1 2 3
**23**	I feel I react too slowly to things that are said to me	0 1 2 3
**24**	I cannot keep an activity going for long	0 1 2 3

UK Meningitis Study Investigators and affiliations (*Northwest sites)

Adedeji Adekola, Mid Yorkshire NHS Hospital Trust; Katharine Ajdukiewicz, North Manchester General Hospital*; David Birkenhead, Calderdale Hospitals Foundation Trust, Tom Blanchard, North Manchester General Hospital*, Antony Cadwgan, University Hospitals of North Midlands; David Chadwick, South Tees Hospitals NHS Foundation Trust; John Cheesbrough, Lancashire Teaching Hospitals*; Richard Cooke, Aintree University Hospitals NHS Trust*; John Croall, Countess of Chester Hospital*; Iain Crossingham, East Lancashire NHS Hospitals*; James Dunbar, Bradford Royal Infirmary; Simon Ellis, Northumbria Healthcare NHS Foundation Trust; Camelia Faris, Wigan, Wrightington and Leigh NHS Foundation Trust*; Peter Flegg, Blackpool Teaching Hospitals NHS Foundation Trust*; Clive Graham, North Cumbria University Hospitals*; Katherine Gray, Lancashire Teaching Hospitals*; Shirley Hammersley, Mid Cheshire NHS Hospitals Foundation Trust*; Kevin Jones, Bolton NHS Foundation Trust*; Matthew Jones, Salford Royal NHS Foundation Trust*; Ildiko Kustos, Countess of Chester Hospital*; Susan Larkin, Aintree University Hospitals NHS Trust*; Karim Mahawish, Warrington and Halton NHS Foundation Trust*; Sarah Maxwell, Stockport NHS Foundation Trust*; Jane Minton, Leeds Teaching Hospitals Trust; Kavya Mohandas, Wirral University Teaching Hospitals NHS Foudnation Trust*; Martin Mostert, Lewisham and Greenwich NHS Trust; Ed Moran, Heart of England NHS Foundation Trust; Christopher Murphy, Fairfield General Hospital*; Monika Pasztor, University Hosptials of Morecombe Bay NHS Foundation Trust*; Hassan Paraiso, Dudley Group NHS Foundation Trust; Nikhil Premchand, Newcastle Hospitals NHS Foundation Trust and Northumbria Healthcare NHS Foundation Trust; Haris Rathur, Tameside Hospital NHS Foundation Trust*; Mark Roberts, University Hospital of South Manchester*; Amy Robinson, Bradford Royal Infirmary; Andrew Rosser, University Hospitals of Leicester NHS Trust; Stefan Schumacher, Bolton NHS Foundation Trust*; Monty Silverdale, East Cheshire NHS Trust*; Philip Stanley, Bradford Royal Infirmary; Neil Todd, York Teaching Hospitals NHS Foundation Trust; Alastair Watt, Northern Devon Healthcare NHS Trust; Martin Wiselka, University Hospitals of Leicester NHS Trust.
